# Intracisternal IGF-1 gene delivery attenuates early anxiety-like behavior but not dopaminergic neurodegeneration in a 6-OHDA rat model of parkinsonism

**DOI:** 10.3389/fnagi.2026.1781503

**Published:** 2026-05-04

**Authors:** Leandro Gabriel Champarini, Macarena Lorena Herrera, Matías Jávega Cometto, Rocío del Valle Bartolozzi, Aracely Janneth Naranjo Viteri, Rosana Crespo, Gastón Diego Calfa, Claudia Beatriz Hereñú

**Affiliations:** IFEC-CONICET, Departamento de Farmacología Otto Orsingher, Facultad de Ciencias Químicas, Universidad Nacional de Córdoba, Haya de la Torre y Medina Allende, Ciudad Universitaria, Córdoba, Argentina

**Keywords:** anxiety, gene therapy, IGF-1, neurotrophic factor, non-motor symptoms, Parkinson’s disease

## Abstract

**Background:**

Parkinson’s disease (PD) is a neurodegenerative disorder characterized by the progressive loss of dopaminergic neurons, leading to a spectrum of motor and non-motor symptoms. In addition to the motor deficits serving as the primary criteria for diagnosis, mood and anxiety disorders also play a significant role in shaping the prognosis and overall disease progression in PD.

**Objectives:**

In this study, our aim was to characterize the progression of anxiety-like behavioral deficits in a rat model of neurotoxicity induced by 6-hydroxydopamine (6-OHDA) and to investigate insulin-like growth factor 1 (IGF-1) potential therapeutic effects on these pathological markers.

**Methods:**

Behavioral changes were evaluated in male Wistar rats at 1, 2, and 3 weeks post-lesion using the elevated plus maze and dark–light box tests. Anxiety-like behaviors emerged as early as week 1 post-lesion and persisted through week 3. Following 6-OHDA infusion, immunohistochemical analysis revealed a decrease in tyrosine hydroxylase (TH) immunoreactivity within the ventral tegmental area (VTA) indicating a partial lesion of the nigrostriatal dopaminergic system. As a therapeutic approach we performed the intracisternal administration of a recombinant adenoviral vector encoding IGF-1 (RAd IGF-1) one week after 6-OHDA-induced neurotoxicity.

**Results:**

At a behavioral level, IGF-1 treatment effectively prevented anxiety-like behavior by the third week of neurotoxicity. Nevertheless, IGF-1 overexpression was not able to modulate VTA dopaminergic neuron loss. These results reveal a dissociation between the behavioral effects of IGF-1 and its impact on dopaminergic neurodegeneration.

**Discussion:**

These findings emphasize time-dependent alterations in anxiety-like behavior and dopaminergic neurodegeneration. In addition, these data support the potential use of IGF-1 as a therapeutic molecule and its novel administrations for future gene transfer interventions, which will shed light on the progressive nature of neurodegenerative mechanisms.

## 1 Introduction

Neuropsychiatric non-motor signs and symptoms (NMS) acquire significant relevance in many individuals diagnosed with Parkinson’s disease (PD) (Weintraub and Mamikonyan, 2019). Furthermore, a series of NMS precedes the typical motor disorder, establishing a clear prodromal stage of the disease (Schapira et al., 2017). Among neuropsychiatric NMS, anxiety-related disorders present the highest incidence in PD, even greater than in any other chronic disease (38% vs. 11%) (Salari et al., 2020). Conversely, individuals diagnosed with an anxiety disorder have an increased risk of developing PD (Pfeiffer, 2016). Anxiety frequently presents as Generalized Anxiety Disorder, but it can also manifest as panic attacks, social phobia, and agoraphobia (Broen et al., 2016), and is often comorbid with depression. Notably, the prevalence of anxiety may be more stable throughout the course of the disease than that of depression (Dlay et al., 2020). Employing a bilateral infusion of 6-OHDA into the dorso-lateral striatum, we have previously reported the presence of NMS, such as early cognitive deficits at 3 weeks of neurotoxicity, before the onset of locomotor deficits (Herrera et al., 2020, 2024a). This experimental design allows us to establish a 3 week-long temporal frame where different behavioral tests enable the study of the presence of different non-motor alterations in this PD model. In the present study, we focus on the anxiety-like behavior that might be associated with this neurodegenerative model.

IGF-1 is considered a multisystem pleiotropic neurotrophic factor (Fernandez and Torres-Aleman, 2012; Nuñez et al., 2023), with different targets that have a positive impact on the brain homeostasis in health and disease (Deak and Sonntag, 2012; Dyer et al., 2016). Clinical studies demonstrate a complex, yet poorly understood, relationship between IGF-1 and the clinical presentation of PD. Studies show that IGF-1 levels are elevated both in serum and cerebrospinal fluid in early phases of the disease (Bernhard et al., 2016). Higher IGF-1 concentrations are associated with better motor and cognitive performance, while lower early IGF-1 levels predict poorer clinical outcomes (Pellecchia et al., 2014; Picillo et al., 2017; Castilla-Cortázar et al., 2020). In relation to PD, Shi et al. reported that individuals with PD who do not exhibit anxiety-related symptoms show higher serum IGF-1 levels compared to those who do present such symptoms (Shi et al., 2023).

We have previously reported that IGF-1 exerts a neuroprotective effect on cognitive deficit, and hinders dopaminergic neurodegeneration after 6-OHDA administration (Herrera et al., 2024a). In this study, we evaluated the onset of anxiety-like behaviors in the 6-OHDA parkinsonism model and we evaluated the effect of IGF-1 overexpression in such impairment. We confirmed that anxiety-like behavior emerges early after administration of 6-OHDA into the dorsal-lateral striatum. This was accompanied by dopaminergic neurodegeneration in the VTA. Intra-cisterna magna delivery of RAd-IGF1 was able to modulate behavioral deficits, although it did not exert a detectable neuroprotective effect. These results support the use of the classical 6-OHDA neurotoxin to study non-motor alterations in animal models of PD. Furthermore, our results provide new insights into the complex relationship of IGF-1 and non-motor symptoms in PD.

## 2 Materials and methods

### 2.1 Animal model

We employed male Wistar rats (60 days old, 280–320 g of weight) born and bred in the animal facility of the Department of Pharmacology Otto Orsingher, Faculty of Chemical Sciences, National University of Córdoba. The animals were kept in optimal temperature conditions (21 ± 2 °C) and controlled light (12 h light/12 h darkness, lights on from 7.00 a.m. to 7.00 p.m.), in groups of 3 animals per Plexiglas cage, with free access to food and water throughout the experiment. All the experiments conducted in this work respected the guidelines for the care and use of laboratory animals provided by the National Institute of Health (NIH), Institute of Laboratory Animal Resources, Guide for the Care and Use of Laboratory Animals (National Academies, USA, 8th edition, 2011). These protocols were approved by the Institutional Committee for the Care and Use of Laboratory Animals (RES #1451/2021) of the Faculty of Chemical Sciences, National University of Córdoba. Taking into account bioethical guidelines, special attention was paid to reducing the number of animals used to the minimum required for statistical precision.

#### 2.1.1 6-OHDA model

To obtain a model of early neurodegeneration with a partial lesion of the nigrostriatal dopaminergic pathway, we followed a previous protocol established in our laboratory (Herrera et al., 2020). Microinjections were bilaterally administered into the dorsolateral striatum using stereotaxic coordinates (Paxinos and Watson, 2007) (AP: +0.2 mm, ML: ±3.5 mm, DV: −4.8 mm from Bregma). Each animal was infused with 2 μL of vehicle solution per hemisphere (saline solution with 0.02 μL) at a flow rate of 0.5 μL/min. A total of 40 μg of the neurotoxin was administered per animal, using a 30G injector connected by polyethylene tubing to a 10 μL Hamilton syringe. An infusion pump was used to ensure administration at the correct volume and flow rate. After the microinjection, the cannula was left at the infusion site for 2 min before being withdrawn to allow for complete drug diffusion. Stereotaxic surgeries were performed under anesthetic conditions: ketamine (55 mg/kg) / xylazine (11 mg/kg, intraperitoneal). After surgery, the animals remained under observation for 24 h in a separate room. They then continued in the post-surgery animal room until the time of the behavioral tests and/or sacrifice.

### 2.2 Behavioral tests

All behavioral assessments were conducted in an acoustically isolated room under controlled lighting and temperature conditions. Experiments were performed between 09:00 and 14:00 h to minimize circadian variability, and all behavioral testing took place between April and October. Animal behavior was recorded using a high-resolution video system, and analyses were performed by an experimenter blinded to treatment groups. Between animals, all apparatuses were thoroughly cleaned with 70% ethanol and dried to remove odor cues and prevent interference across trials. Motor performance in this 6-OHDA model has been extensively characterized by our group under the same experimental conditions. In our previous studies, we performed locomotor activity tests and demonstrated that, at the early time points evaluated in the present work, 6-OHDA treated rats do not exhibit detectable motor impairments (Herrera et al., 2020, 2024a).

#### 2.2.1 Elevated plus maze (EPM)

These evaluations were performed in an acoustically isolated behavioral room at 7, 14, and 21 days after stereotaxic surgery. For each of the different time points, different sets of animals were used to avoid repeating exposure to the same behavioral paradigm. The EPM consisted of two opposing open arms (50 × 10 cm) and two opposing closed arms (50 × 10 cm), with walls 40 cm high and no roof, with a central platform measuring 10 × 10 cm. The entire apparatus was elevated 50 cm off the ground. Animals were habituated to the room for 30 min before being tested. During the test, the animal was placed on the central platform facing an open arm and was allowed to explore freely for 5 min. The percentage of time spent in the open arms, the number of entries into the open arms and closed arms were recorded. The number of entries into the closed arms is related to locomotor activity and represents a measure of it (Walf and Frye, 2007). After testing each animal, the apparatus was sanitized with a 70% alcohol solution and dried before the next animal was evaluated.

#### 2.2.2 Dark/light box test (DLB)

The test apparatus consisted of a dark, safe compartment (completely sealed, with dark walls and a dark rubber floor) and a lit, aversive compartment (white walls, a transparent lid, and a white acrylic floor). The illuminated compartment is lit by a lamp (300–350 lux). Both compartments are connected by an opening in the shared wall. At the beginning of the test, this opening is covered with a sliding acrylic lid. The animal is placed inside the dark compartment, and after 20 s, the gate is opened so the animal can freely explore the apparatus. This exploration is recorded for 5 min. The variables to consider are time spent in the lit compartment, and the number of visits to the lit compartment. After testing each animal, the apparatus was sanitized with a 70% alcohol solution and dried before the next animal was evaluated.

### 2.3 IGF-1 overexpression

The recombinant adenoviral vectors (RAds) were previously constructed in our laboratory (Hereñú et al., 2007) as carriers to deliver either the cDNA of IGF-1 gene (RAd-IGF1) or the red fluorescent protein from Discosoma sp. DsRed (RAd-DsRed), as a control group.

#### 2.3.1 Intracisternal (ICM) administration of adenoviral vectors

In a different set of animals, we performed the intra-cisterna magna (ICM) administration of recombinant adenoviral vectors, one week after 6-OHDA administration. For this procedure, each animal was anesthetized with an intraperitoneal dose of ketamine (55 mg/kg) and xylazine (11 mg/kg). After confirming full anesthesia, the animals were placed in a stereotaxic apparatus, and the head was then tilted downward to an approximate 110° angle from the horizontal axis. An insulin syringe (BD Insulin Syringe 8 mm x 0.3 mm) was loaded with 10ul of the vector-containing solution. The needle was introduced vertically, and approximately 20 μL of cerebrospinal fluid (CSF) was withdrawn to confirm proper access to the cisternal cavity. The CSF-vector solution was then slowly and steadily re-infused. After waiting 10 s to prevent solution reflux, the syringe was carefully withdrawn. The animal’s head was then repositioned to a 0° angle with respect to the horizontal axis. We ensured the animal was breathing correctly before returning it to its housing. Post-procedural monitoring was performed for several days to ensure the animals’ well-being.

### 2.4 Immunofluorescence

Animals were placed under deep anesthesia and perfused with phosphate-buffered paraformaldehyde 4%, (pH 7.4) fixative. The brains were removed, stored in paraformaldehyde 4%, (pH 7.4) overnight at 4 °C, and were kept in cryoprotective solution at −20 °C until use. For immunohistochemical assessment, brains were cut coronally into 25 μm-thick sections with a cryostat microtome (Leica). Sections were washed three times, for 5 min each, with 0.01 M PBS (pH 7.4). Then they were incubated in a blocking solution containing 5% normal goat serum (NGS, Serendipia Lab, Santa Fe, Argentina) for 2 h with gentle agitation to block non-specific binding sites. The sections were then incubated with the primary antibody for TH (Dilution 1:2500, Thermo Fisher Scientific Cat # OPA1-04050) for 12 h at room temperature. After this incubation, the sections were washed again with 0.01 M PBS and incubated for 2 h at room temperature with the appropriate fluorescent anti-rabbit secondary antibody (dilution 1:500, abcam Cat# ab150077). After three consecutive washes with 0.01 M PBS, the sections were incubated with DAPI for 30 min, followed by two more washes. Finally, the sections were mounted on gelatin-coated slides and covered with FluorSave mounting medium (Sigma-Aldrich). Representative images of the experimental groups were acquired using a computerized system with an Olympus FV1200 confocal microscope. The images were saved in TIFF format (1,392 × 1,040 pixels). During the image processing, three sections from each rat were analyzed. The number of TH + cells in the VTA or fluorescence intensity in the basolateral amygdala (BLA) was counted for the analysis. For each rat, measurements were taken in both hemispheres across different sections to provide relative data for various experimental conditions.

### 2.5 Statistical analysis

Statistical analysis was performed using GraphPad Prism 10.0.3 software (Windows version, GraphPad Software, Inc., San Diego, CA, USA). Comparisons between groups were made using Student’s *T*-test, One-way or two-way Analysis of Variance (ANOVA). When a significant effect was detected by ANOVA, Tukey’s *post hoc* test was applied for multiple comparisons. A value of *p* < 0.05 was considered statistically significant. The specific statistical tests utilized for each comparison, along with the reported significance, are detailed in the figure legends.

## 3 Results

### 3.1 Bilateral 6-OHDA infusion induces a progressive emergence of anxiety-like behavior in the elevated plus maze test

**Figure 1:**
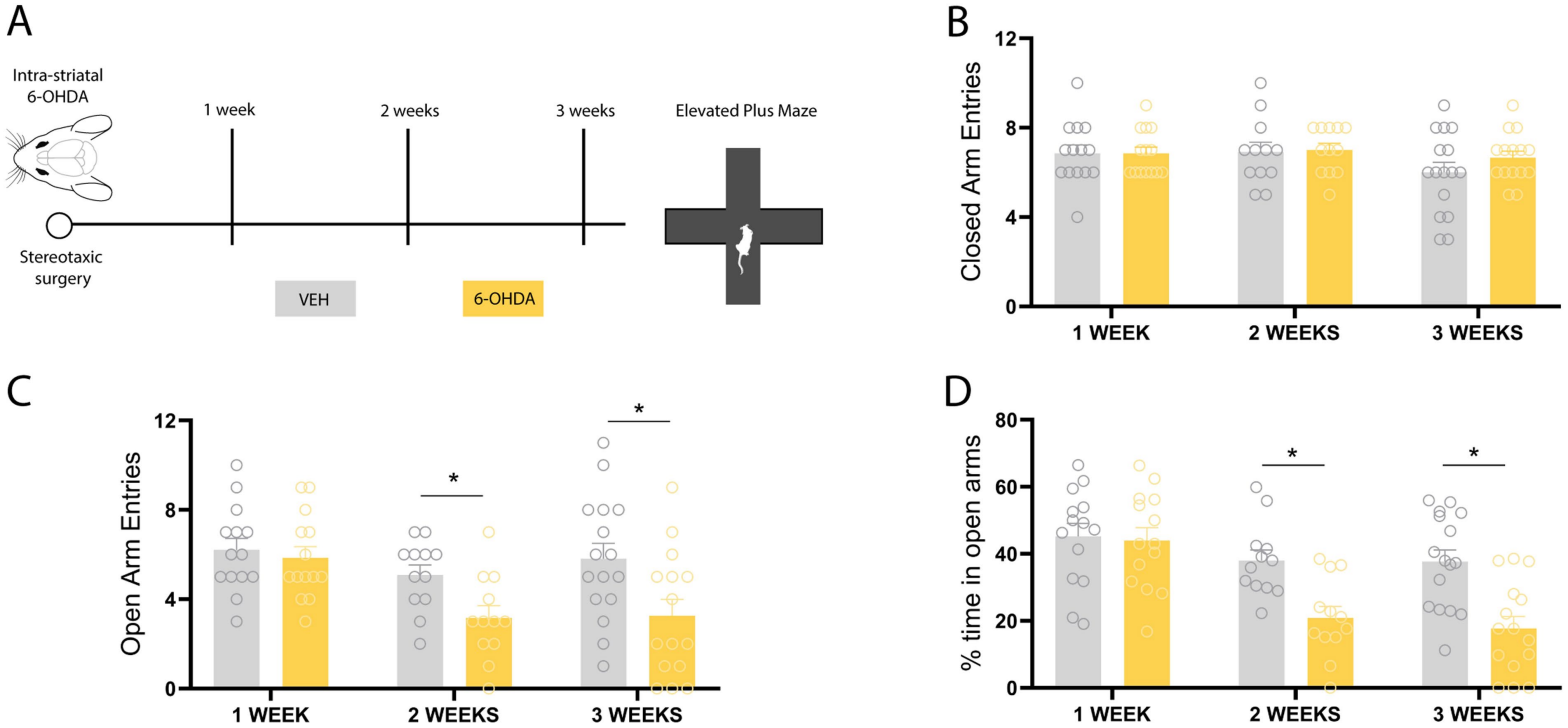
Behavioral assessment of anxiety-like behavior after intrastriatal administration of 6-OHDA in the elevated plus maze. **(A)** Schematic of the experimental design. **(B)** Entries into the closed arms: 1 week (*t* = 0.00; *p* > 0.99), 2 weeks (*t* = 0.1576; *p* = 0.8762), and 3 weeks (*t* = 1.217; *p* = 0.233) after 6-OHDA injection. **(C)** Percentage of time spent in the open arms: 1 week (*t* = 0.23; *p* = 0.82), 2 weeks (*t* = 3.68; *p* = 0.0013), and 3 weeks (*t* = 3.99; *p* = 0.0004) after 6-OHDA injection. **(D)** Entries into the open arms: 1 week (*t* = 0.5; *p* = 0.62), 2 weeks (*t* = 2.699; *p* = 0.0131), and 3 weeks (*t* = 2.54; *p* = 0.017) after 6-OHDA injection. *n* = 16–15 animals. Student’s *t*-test. All data are presented as mean ± SEM. *Indicates a statistically significant difference with a *p*-value less than 0.05.

To begin assessing the development of anxiety-like behavior in 6-OHDA treated animals, we performed the EPM. Based on our previous findings indicating that locomotor impairments under these conditions emerge at the fourth week following 6-OHDA infusion (Herrera et al., 2020), anxiety-like behavior was evaluated at early post-infusion time points, prior to the onset of motor deficits (Figure 1A). This experimental design ensured that locomotor performance did not confound the interpretation of EPM outcomes. 21 days after bilateral infusion of 6-OHDA into the dorsolateral striatum, no differences were observed in closed arm entries (Figure 1B) compared to the control group (*p* = 0.2333; *t* = 1.217). However, we observed a significant decrease in the number of entries to the open arms (Figure 1C) in 6-OHDA animals compared to the control group (*p* = 0.0167; *t* = 2.541). Along with this, we assessed the percentage of the time spent in the open arms (Figure 1D), where the 6-OHDA group spent 17.78% of the total test time in the open arms, compared to the 37.69% of control animals (*p* = 0.0004; *t* = 3.990). These results indicate that 21 days after the infusion of the 6-OHDA neurotoxin, the animals exhibit an anxiety-like behavior in the EPM paradigm. Furthermore, no differences were observed regarding the number of entries into the closed arms, which validates the use of this test in the model, as it suggests no locomotor deficit that would affect the animal’s performance in the apparatus. Following the observation of anxiety-like behavior in the model three weeks post-surgery, we set to temporally delimit the onset of this behavioral phenotype in the EPM. Unlike the first group of animals, behavior in the EPM was evaluated 14 days after the neurotoxin or vehicle infusion. Regarding the number of entries into the closed arms (Figure 1B), no differences were observed between the two groups (*p* = 0.8762; *t* = 0.1576). In terms of the number of entries into the open arms (Figure 1C), the 6-OHDA animals reduced the number of entries than the control group 14 days post-surgery (*p* = 0.0131; *t* = 2.699). The percentage of time spent in the open arms (Figure 1D) was lower in the group of animals that received 6-OHDA compared to the control group. This difference was found to be statistically significant as measured by the Student’s *t*-test (*p* = 0.0013; *t* = 3.682). To further delimit the appearance of the anxious-like phenotype, the same bilateral 6-OHDA infusion protocol was performed, and the EPM test was subsequently conducted 7 days post-surgery. Similar to the observations 21 and 14 days post-surgery, 7 days later the animals showed no difference in the number of entries into the closed arms (Figure 1B) (*p* > 0.999; *t* = 0.00). Regarding the number of entries into the open arms (Figure 1C), no significant differences were observed between the animal groups (*p* = 0.6235; *t* = 0.4968). With respect to the variable of time spent in the open arms (Figure 1D), no significant differences were observed between both groups (*p* = 0.8228; *t* = 0.2262). The 6-OHDA group spent an average of 43.98% of the time in the open arms, and the control group 45.21%. A one-way ANOVA comparing all experimental groups showed no significant differences among vehicle-treated animals, suggesting that EPM performance was not affected by time post-surgery in control conditions. Together, these results suggest the presence of anxiety-like behavior after bilateral 6-OHDA in the absence of locomotor impairment, as evaluated by the EPM test.

### 3.2 Dark/light box test revealed an earlier anxiogenic-like effect of 6-OHDA starting 7 days post-lesion

**Figure 2:**
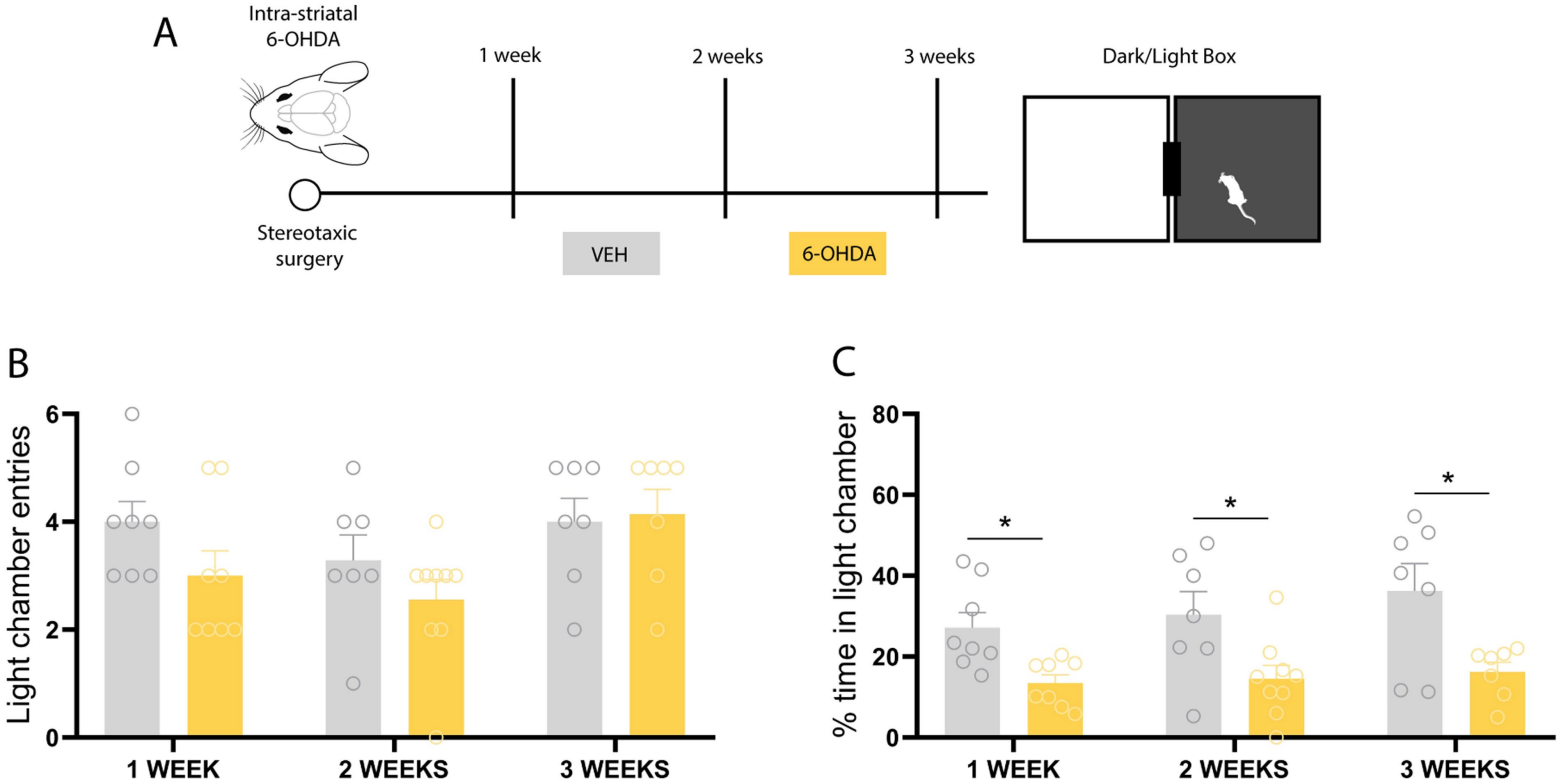
Behavioral assessment of anxiety-like behavior in the dark/light test. **(A)** Schematic of the experimental design. **(B)** Entries into the illuminated compartment: 1 week (*t* = 1.673; *p* = 0.116), 2 weeks (*t* = 1.223; *p* = 0.2415), and 3 weeks (*t* = 0.2255; *p* = 0.8254) after 6-OHDA injection. **(C)** Time spent in the illuminated compartment: 1 week (*t* = 3.208; *p* = 0.0063), 2 weeks (*t* = 2.540; *p* = 0.0236), and 3 weeks (*t* = 2.783; *p* = 0.0166) after 6-OHDA injection. *n* = 7–8 animals. Student’s *t*-test. All data are presented as mean ± SEM. *Indicates a statistically significant difference with a *p*-value less than 0.05.

To further characterize the anxiety-like behavior observed in this prodromal phase of the model, we decided to assess this behavior employing the DLB test. Consistent with the experimental design for the EPM evaluation, we began with a group of animals that received an infusion of 6-OHDA or VEH and were evaluated on the DLB test after 21 days (Figure 2A). Regarding the number of entries into the illuminated compartment (Figure 2B), no differences were observed between the animals that received the bilateral 6-OHDA infusion compared to the vehicle group (*p* = 0.8254; *t* = 0.2255). Conversely, the time spent in the illuminated compartment was significantly lower in the 6-OHDA animals (Figure 2C), who presented a 16.24% permanence, compared to the 36.24% permanence of the vehicle group (*p* = 0.0166; *t* = 2.783). In summary, these results demonstrate that 21 days after the induction of the neurodegeneration model, the animals display an anxiety-like behavior in the DLB test. Similarly to the EPM, we sought to temporally delimit the onset of the anxiety-like phenotype in the DLB test. Thus, a second group of animals was tested in the DLB test 14 days post-surgery. Regarding the number of entries into the illuminated compartment (Figure 2B), no differences were observed between the animals that received the bilateral 6-OHDA infusion compared to the vehicle group (*p* = 0.2415; *t* = 1.223). Concerning the time spent in the illuminated compartment, the 6-OHDA group remained in it significantly less time than the vehicle group (Figure 2C) (*p* = 0.0236; *t* = 2.540). Finally, a third group of animals received the 6-OHDA or vehicle infusion, and their DLB behavior was recorded 7 days post-surgery. No significant differences were observed regarding the number of entries into the illuminated compartment (Figure 2B) (*p* = 0.1165; *t* = 1.673). However, the time spent in the illuminated compartment was significantly lower in the 6-OHDA animals (Figure 2C) (*p* = 0.0063; *t* = 3.208). A one-way ANOVA comparing all experimental groups showed no significant differences among vehicle-treated animals, suggesting that DLB performance was not affected by time post-surgery in control conditions. Consistent with the results observed in the EPM, these results suggest that anxiety-like behavior is also observed when employing the DLB test and persists at 2 and 3 weeks following neurotoxin infusion. Notably, this phenotype is evidenced earlier by the DLB, as it is detectable one week post-surgery.

### 3.3 Intracisternal administration of RAd-IGF1 blunts 6-OHDA-induced anxiety-like behavior in the DLB

**Figure 3:**
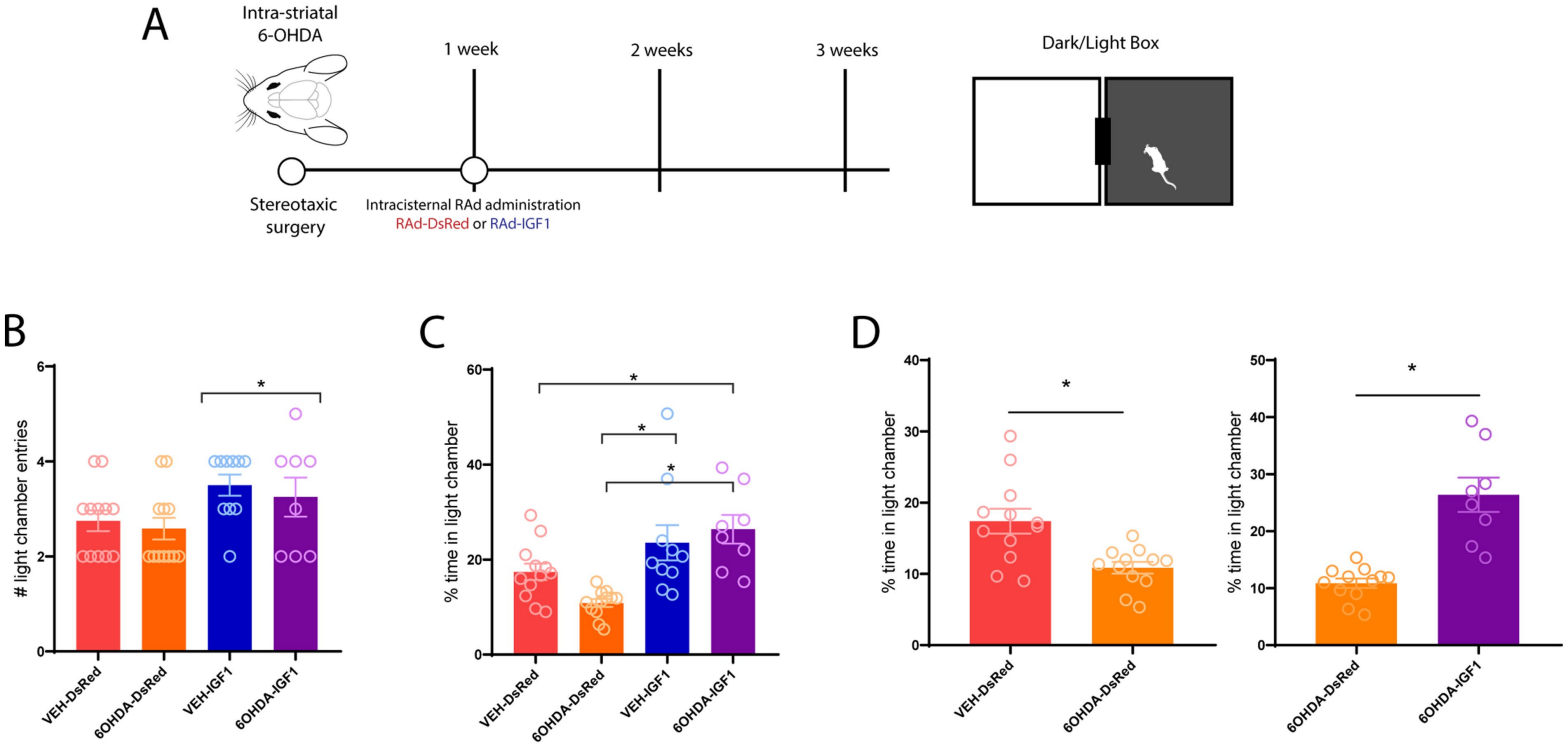
Effect of intracisternal administration of RAd-IGF-1 on 6-OHDA-induced anxiety-like behavior. **(A)** Schematic of the experimental design. **(B)** Entries into the illuminated compartment [*F*(1, 36) = 5,672; *p* = 0.0226]. **(C)** Time spent in the illuminated compartment [*F*(1,41) = 20.20; *p* < 0.0001]. **(D)** Student *T*-test assessing difference between gene therapy groups: VEH-DsRed vs. VEH-IGF1: *p* = 0.0025 6OHDA-DsRed vs. 6OHDA-IGF1 *p* < 0.0001. VEH-DsRed, VEH-IGF1, 6OHDA-DsRed, 6OHDA-IGF1 sample sizes of: 12, 10, 12, and 9 animals, respectively. The data was analyzed using a two-way ANOVA and is presented as the mean value ± SEM. *Indicates a statistically significant difference with a *p*-value less than 0.05.

It has been demonstrated that IGF-1 acts as a neurotrophic factor and neuromodulator with an influence on various behaviors (Champarini et al., 2022; Herrera et al., 2024a). Consequently, we decided to evaluate the effect of IGF-1 overexpression in the anxiety-like behavior induced by the parkinsonism model. One week after intra-CPu infusion of 6-OHDA or vehicle by stereotaxic surgery, animals received intracisternal (ICM) administration of RAd-IGF1 or RAd-DsRed (Figure 3A). This allowed for a 14-day long expression period of the cDNA from the viral vectors (Herenu et al., 2009). Subsequently, anxiety-like behavior was assessed using the DLB test. Regarding the number of entries into the light compartment, a significant vector effect was observed [*F*(1, 36) = 5,672; *p* = 0.0226], where animals that received RAd-IGF1 administration showed an increased number of entries to the illuminated compartment (Figure 3B). With respect to the time spent in the illuminated compartment, the two-way analysis of variance demonstrated a significant vector effect [*F*(1,41) = 20.20; *p* < 0.0001], where animals that received RAd-IGF1 administration showed an increased percentage of time spent in the illuminated compartment (Figure 3C). Conversely, no model effect was observed [*F*(1,41) = 0.3234; *p* = 0.5727], nor was an interaction effect observed [*F*(1,41) = 3.282; *p* = 0.0774]. Tukey’s *post hoc* analysis demonstrated that the 6-OHDA-IGF1 animal group remained in the illuminated compartment longer than the 6-OHDA-DsRed group (*p* = 0.0003). In contrast, no differences were observed in the vehicle group between the different vector treatments (*p* = 0.2460). These results suggest that overexpression of IGF-1 leads to an anxiolytic-like behavior as observed in the DLB. Further statistical comparison between groups showed that this difference was more pronounced between the 6-OHDA treated animals (Figure 3D).

### 3.4 ICM RAd-IGF1 administration could not prevent dopaminergic neuron loss in the ventral VTA

**Figure 4:**
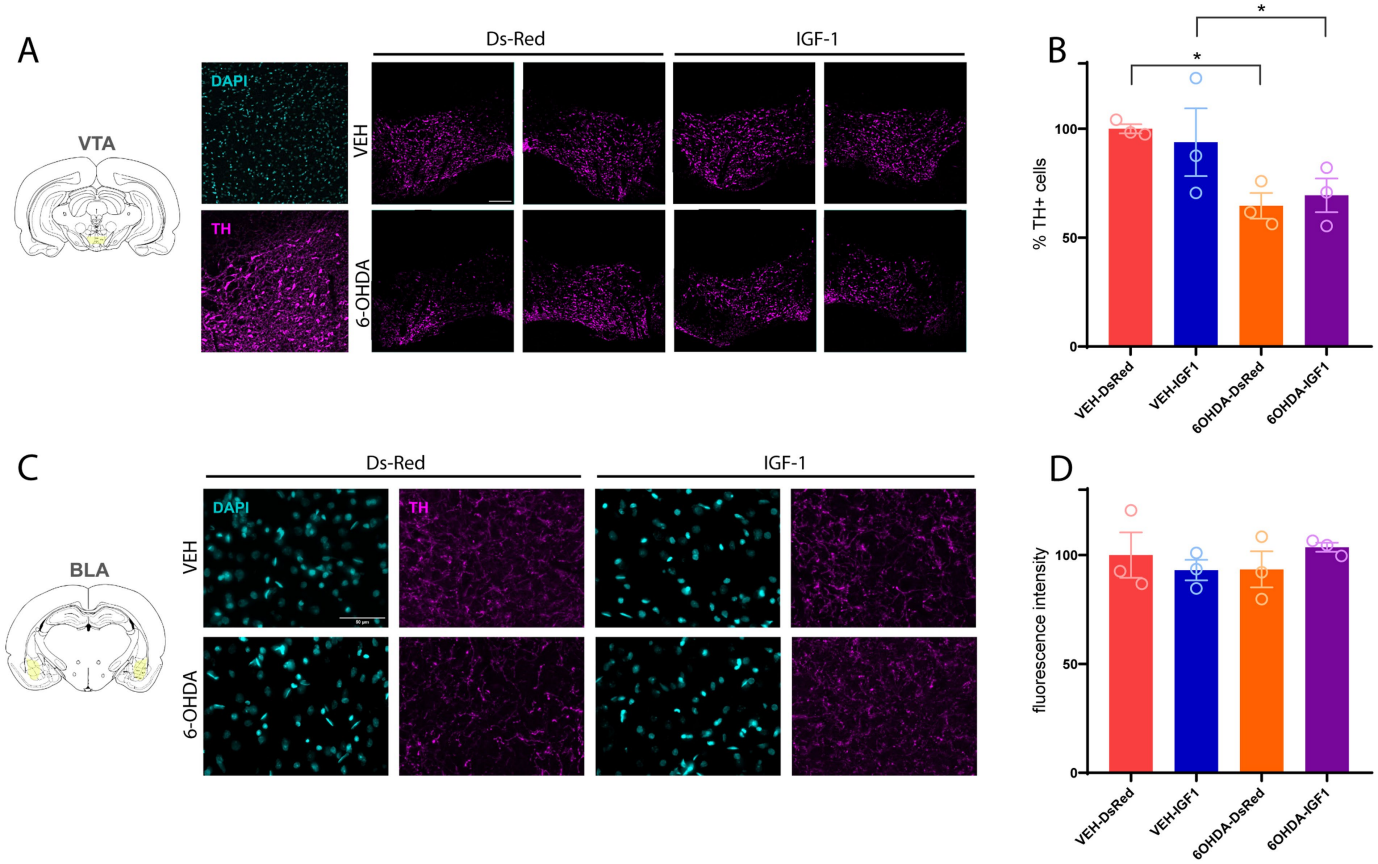
Effect of intracisternal administration of RAD-IGF1 on 6-OHDA-induced dopaminergic neurodegeneration. **(A)** Representative images of dopaminergic cell bodies of the VTA at 10× magnification. Scale bar: 200 μm. **(B)** Percentage of TH-positive (TH+) cells in the VTA relative to the control group. **(C)** Representative images of dopaminergic fibers of the BLA at 63x magnification. Scale bar: 50 μm. **(D)** Percentage of TH-positive (TH+) immunofluorescence intensity of BLA dopaminergic fibers relative to the control group. The data was analyzed using a two-way ANOVA and is shown as the mean value ± SEM. *Indicates that a result is statistically significant, with a *p*-value less than 0.05.

We have previously reported that 6-OHDA infusion leads to a decrease in dopaminergic cell bodies in the substantia nigra and in dopaminergic projections to the striatum (Herrera et al., 2020, 2024a). Although this intrastriatal lesion paradigm primarily disrupts the nigrostriatal pathway, partial involvement of dopaminergic neurons in the VTA has also been described (Issy et al., 2015; Masini et al., 2021). In addition to the nucleus accumbens and the prefrontal cortex, dopaminergic neurons from the VTA project to the amygdala subnuclei, including the BLA (Lutas et al., 2019; Tang et al., 2020), a structure crucial in anxiety processes (LeDoux et al., 1990; Kienast et al., 2008). We sought to determine the effect of RAd-IGF1 administration and 6-OHDA on the dopaminergic neurodegeneration in the VTA cell bodies. To analyze the effect of gene therapy with RAd-IGF1, we proceeded to quantify dopaminergic neurodegeneration by means of TH immunostaining in the VTA. The two-way ANOVA test demonstrated a significant model effect [*F*(1,8) = 10.48; *p* = 0.0119], where the 6-OHDA infusion caused a significant decrease in the percentage of dopaminergic neurons in the VTA (Figures 4A,B). No IGF-1 overexpression effect was observed [*F*(1,8) = 0.005368; *p* = 0.9434], In this regard, intracisternal administration of the vector had no effect on the dopaminergic neurodegeneration caused by 6-OHDA in the VTA. We also observed TH immunofluorescence in the BLA to assess dopaminergic fiber density in this region. In contrast to the effects observed in the VTA, TH immunostaining in the BLA was not affected by the neurodegeneration model (Figures 4C,D) [*F*(1, 8) = 0.07704; *p* = 0.7884], vector administration [*F*(1, 8) = 0.05189; *p* = 0.8255], or the interaction between both factors [*F*(1, 8) = 1,432; *p* = 0.2656]. Although ICM RAd-IGF1 administration was able to elicit an anxiolytic-like effect in behavior, it did not prevent 6-OHDA-induced dopaminergic neurodegeneration in the VTA.

## 4 Discussion

The present study demonstrates that this 6-OHDA parkinsonism model induces an early anxiogenic phenotype, prior to the onset of motor impairments. The early onset of this non-motor symptom adds to the early appearance of previously reported cognitive deficits in this model (Herrera et al., 2020, 2024a). This finding aligns with clinical observations indicating that non-motor symptoms, including anxiety, frequently precede diagnosis and significantly worsen the quality of life in patients with PD. Our behavioral characterization revealed that anxiety-like behavior becomes detectable as early as one week after 6-OHDA lesion with the DLB test, and subsequently persists at two and 3 weeks in both the EPM and DLB paradigms. This temporal difference in paradigm sensitivity was central to the selection of the behavioral assay used to evaluate the effect of IGF-1, since vectors were administered one week post-lesion, coinciding with the earliest detectable onset of anxiety-like behavior. At this time window, the EPM had not yet shown sensitivity to 6-OHDA-induced anxiety, limiting its utility in detecting a potential reversing effect of IGF-1. Although both tests are widely employed to assess anxiety-related behavior (Riebe and Wotjak, 2012), differences between paradigms are not uncommon and may reflect their sensitivity to different aspects of anxiety-like behaviors in rodents (e.g., open-space aversion vs. light aversion) (Chaouloff et al., 1997; Miyakawa et al., 2003; Ramos et al., 2008; Angulo et al., 2025). Our results are consistent with previous studies showing that 6-OHDA induces an anxiogenic-like state in rodents (Tadaiesky et al., 2008; Chen et al., 2011; Bonito-Oliva et al., 2014; Beppe et al., 2015; Ferrazzo et al., 2019; Vieira et al., 2019). Importantly, behavioral evaluations were conducted within a time window in which locomotor performance remains preserved in this model, as previously characterized by our group (Herrera et al., 2020, 2024a). Consistent with this, in the present study the number of closed arm entries in the EPM, commonly used as an index of general locomotor activity, did not differ between experimental groups (Figure 1B). The preservation of locomotor activity at the time points analyzed minimizes potential confounding effects of motor impairment on the interpretation of anxiety-like behavior, which is particularly relevant given that alterations in motor performance may mimic or mask anxiety-related responses (Acevedo et al., 2014).

We next evaluated the therapeutic potential of IGF-1 overexpression delivered through recombinant adenoviral vectors. We optimized an intrathecal (ICM) approach, which proved effective for achieving widespread distribution within the ventricular system (Supplementary Figures 1, 2). Previous work from our group has shown that following intraventricular RAd-IGF1 administration, CSF IGF-1 levels reach maximal expression approximately five days after injection and remain elevated for around 20 days before gradually declining (Herenu et al., 2009), ensuring robust IGF-1 expression at the time of behavioral assessment. ICM administration of RAd-IGF1 produced an anxiolytic-like effect in 6-OHDA-lesioned animals, reflected by increased time spent in the illuminated compartment at 3 weeks after the lesion. This effect was specific to the lesioned groups, as no differences were observed between VEH-DsRed and VEH-IGF1 animals. These findings are particularly promising given that vector administration overlapped with the onset of anxiety-like behavior, which emerged one week after 6-OHDA injection. While further studies will be necessary to determine the persistence of this behavioral effect, longer follow-up evaluations in this model are limited by the progressive emergence of locomotor impairments after 4 weeks of 6-OHDA lesion, potentially undermining the detection of anxiety-related behavioral outcomes.

At the neurobiological level, we observed a significant loss of dopaminergic neurons in the VTA at 3 weeks post-lesion. These results complement previous results of our group, where we reported dopaminergic neuron loss in the substantia nigra pars compacta (SNpc), CPu, dorsal hippocampus and prefrontal cortex, employing the same 6-OHDA infusion protocol used in the present study (Herrera et al., 2024a).

The mesocorticolimbic dopaminergic system, including projections to the nucleus accumbens, prefrontal cortex, and BLA, is implicated in anxiety and affective regulation (van der Wee et al., 2008; Zweifel et al., 2011; Russo and Nestler, 2013; Lutas et al., 2019; Tang et al., 2020). Reduced VTA → BLA activity has been causally associated with anxiety-like states (Morel et al., 2022), suggesting that degeneration in this pathway may underlie the behavioral alterations observed. In PD pathophysiology, degeneration is most severe in the SNpc, leading to the defining motor symptoms. The VTA is generally considered relatively spared, yet its impairment is functionally significant, underpinning NMS such as motivational deficits and depression (Drui et al., 2014). Thus, comprehensive modeling of PD models requires assessment of both midbrain nuclei. Retrograde transport of 6-OHDA from the striatum to the midbrain nuclei results in a cascade of toxic events. Quantitative assessments in rat models confirm that while the SNpc is more severely affected, the VTA experiences significant and measurable neurodegeneration following intrastriatal 6-OHDA administration (Haas et al., 2016; Masini et al., 2021; Mendes-Pinheiro et al., 2021). Our results confirm that VTA neurons are also vulnerable to the retrograde cytotoxic lesion initiated at the dorsolateral striatal terminals. Notably, 6-OHDA is preferentially taken up by dopamine transporters, however, partial involvement of noradrenergic or serotonergic systems cannot be excluded (Bezard et al., 2013; Vieira et al., 2019), further contributing to the complex neurochemical alterations driving anxiety in PD models. These results prompt us to keep studying the mechanisms underlying anxiety-like behaviors in the 6-OHDA model assessing how other neurotransmitter systems may be involved.

IGF-1 emerges as a particularly strong candidate for dopaminergic neuroprotection because it targets multiple mechanisms relevant to the vulnerability of the dopaminergic system. Previous evidence indicates that IGF-1 receptors (IGF-1R) are expressed in midbrain dopaminergic neurons, including a substantial proportion of tyrosine hydroxylase positive neurons in the VTA (Pristera et al., 2019; Zegarra-Valdivia et al., 2026). Moreover, IGF-1R is widely distributed across multiple neuronal populations in limbic structures, including the BLA, where IGF-1 signaling has been implicated in synaptic modulation and fear-related plasticity (Maglio et al., 2021; Champarini et al., 2022). The high expression of IGF-1R in dopaminergic neurons makes them especially sensitive to IGF-1 mediated neurotrophic effects (Mashayekhi et al., 2010). IGF-1 promotes dopaminergic neuron survival and differentiation, increases dopamine uptake, and prevents programmed cell death *in vitro* and *in vivo* (Quesada and Micevych, 2004; Ebert et al., 2008). When IGF-1 binds its cognate receptor, it activates the PI3K/Akt pathway, a major pro-survival cascade impaired in PD models (Malagelada et al., 2008; Akundi et al., 2012). This activation inhibits GSK-3β, suppresses caspase activity, stabilizes mitochondrial function, and promotes CREB-dependent neuronal maintenance (Quesada et al., 2008; Sun et al., 2010). IGF-1 also counteracts oxidative stress and neuroinflammation, two central drivers of dopaminergic degeneration, by reducing ROS production and regulating microglial activation toward a neuroprotective phenotype (Rodriguez-Perez et al., 2016; Herrera et al., 2024b). Consistent with these mechanisms, IGF-1 protects against dopaminergic cell loss in multiple PD toxin models, including 6-OHDA, as we have previously reported (Herrera et al., 2024a). Furthermore, DA neurons synthesize and secrete their own IGF-1, which is required for maintaining firing properties, tyrosine hydroxylase phosphorylation, dopamine synthesis, and release (Pristera et al., 2019). Clinical studies also show increased CSF and serum IGF-1 levels in early PD, suggesting an endogenous compensatory neuroprotective response (Picillo et al., 2013; Pellecchia et al., 2014). We have previously shown that the overexpression of IGF-1 employing RAds has been successful at providing dopaminergic neuroprotection (Hereñú et al., 2007; Herrera et al., 2024a). Despite these well-documented neuroprotective actions, IGF-1 overexpression in the present study did not prevent dopaminergic neuronal loss in the VTA, as indicated by TH immunostaining, and no effect of 6-OHDA nor IGF-1 was observed in the BLA. This apparent dissociation between behavioral and neuroprotection effects, suggests that whether this effect is related to dopaminergic system alteration, IGF-1 anxiolytic-like effect could arise from functional modulation of dopaminergic neurotransmission rather than structural neuroprotection. Indeed, IGF-1 has been shown to influence dopaminergic release to the amygdala and striatum (de Oliveira et al., 2014; Pristera et al., 2019). It is also worth noting that in several studies reporting dopaminergic neuroprotection by IGF-1, the peptide was administered prior to or concomitantly with the lesion, conditions under which its effects are more appropriately described as protective. In contrast, the aim of the present work was to evaluate the restorative potential of IGF-1 when administered after the onset of the behavioral alterations. Within this experimental framework, the absence of a detectable effect on VTA dopaminergic neurodegeneration may therefore reflect the more challenging therapeutic context in which IGF-1 was delivered after toxin exposure.

IGF-1 receptors are widely expressed throughout the brain and are present in multiple cell types, including neurons and glial cells (Fernandez and Torres-Aleman, 2012). This widespread distribution highlights the pleiotropic nature of the action of IGF-1 in the CNS. Thus, mechanisms beyond dopaminergic modulation could be underlying the effect observed in anxiety-like behavior. In this regard, IGF-1 has been reported to exert anxiolytic actions through the regulation of mineralocorticoid receptors in the hippocampus (Baldini et al., 2013). In addition, IGF-1 is known to modulate microglial and astrocytic inflammatory responses (Ferger et al., 2010; Bellini et al., 2011; Park et al., 2011; Grinberg et al., 2013; Lee and Tansey, 2013; Dominguez-Meijide et al., 2017; Wang et al., 2023). Our group has previously observed IGF-1-induced modulation of astrocytic and microglia morphology and inflammatory phenotypes in 6-OHDA-treated animals (Herrera et al., 2024a). Our findings align with and extend previous work from our group showing that IGF-1 gene therapy can influence multiple CNS processes. Indeed, IGF-1 delivery has been shown to attenuate microglial reactivity following CNS injury (Herrera et al., 2021), ameliorate cognitive impairments induced by 6-OHDA (Herrera et al., 2024a), and alter microglial phenotype in aged rats (Falomir-Lockhart et al., 2019).

Interestingly, therapeutic benefits have also been observed with IGF-1 derived peptides such as Trofinetide, a synthetic analog of the IGF-1 tripeptide Gly-Pro-Glu, which has demonstrated clinical efficacy in Rett syndrome and has been approved by the FDA for its treatment (Mullard, 2024). Notably, although its therapeutic effects are well established, the precise mechanisms underlying its action remain incompletely understood (Yu et al., 2026). These results highlight the need to investigate the different mechanisms of IGF-1 to better understand the basis of its therapeutic effects. However, the present study did not directly evaluate these pathways, which represents an important limitation to be addressed in future work.

Together, these findings highlight the value of the 6-OHDA model for studying non-motor symptoms of PD (Prates-Rodrigues et al., 2025) and identifying IGF-1 as a promising candidate for modulating early affective disturbances associated with dopaminergic dysfunction. The observation that IGF-1 improved anxiety-like behavior despite persistent dopaminergic loss in the VTA underscores the need to further dissect the mechanisms underlying IGF-1 mediated modulation of anxiety-related responses. In this sense, the study of this neurotrophic factor signaling presents a future and interesting paradigm to keep delving into the development of promising approaches involving IGF-1 gene therapy.

## Data Availability

The raw data supporting the conclusions of this article will be made available by the authors, without undue reservation.

## Supplementary material

SUPPLEMENTARY FIGURE 1 Assessment of expression of RAd-DsRed vector along the ventricular system after intracisternal administration. **(A)** Schematic of the experimental design. Two month old rats were intracisternally administered with RAd-DsRed adenoviral vector. After one week, rats were perfused and we assessed the distribution of the expression of DsRed fluorescent protein (Ex λ 583 nm) along the ventricular system. **(B)** 1, 2, 3 and 4 shows representative images of the rat ventricular system obtained from the Waxholm Space atlas of the Sprague Dawley rat brain (Papp et al., 2014). In panel **(B)**, intra-CPu shows a representative image of the expression of the vector RAd-DsRed locally injected into the striatum. Panel **(B)** 1’, 2’, 3’ and 4’ show our representative images of the expression of DsRed fluorescent protein along the ventricular system. Aq, Aqueduct; 4V, 4th ventricle; 3V, 3rd ventricle; LV, lateral ventricle.

SUPPLEMENTARY FIGURE 2 IGF-1 overexpression assessment. **(A)** Schematic of the experimental design. IGF-1 concentrations were determined using a commercially available electrochemiluminescence immunoassay (ECLIA) (Elecsys IGF-1, Roche Diagnostics), according to the manufacturer’s instructions. The assay is based on a sandwich immunoassay principle and includes an initial acidification step to dissociate IGF-1 from its binding proteins, followed by the formation of a sandwich complex using a biotinylated monoclonal anti-IGF-1 antibody and a ruthenium-labeled monoclonal anti-IGF-1 antibody. The immune complexes are captured on streptavidin-coated microparticles and quantified by electrochemiluminescence detection. Cerebrospinal fluid samples (CSF) from rats were obtained according to standard laboratory procedures and centrifuged prior to analysis when necessary. Samples were maintained at −20 to −25 °C until IGF-1 quantification. **(B)** IGF-1 levels in rat CSF. Due to limited sample volume, cerebrospinal fluid samples from animals belonging to the same experimental group were pooled prior to analysis (number of animals per group: VEH-DsRed = 4, VEH-IGF1 = 1, 6OHDA-DsRed = 3, 6OHDA-IGF1 = 4). Concentrations were automatically calculated by the analyzer using a two-point calibration curve and a lot-specific master curve.
