# Supplementary figures and images for: Intracisternal IGF-1 gene delivery attenuates early anxiety-like behavior but not dopaminergic neurodegeneration in a 6-OHDA rat model of parkinsonism
Source: Front Aging Neurosci. 2026 May 4;18:1781503. doi: 10.3389/fnagi.2026.1781503 (PMC13182658; doi:10.3389/fnagi.2026.1781503)

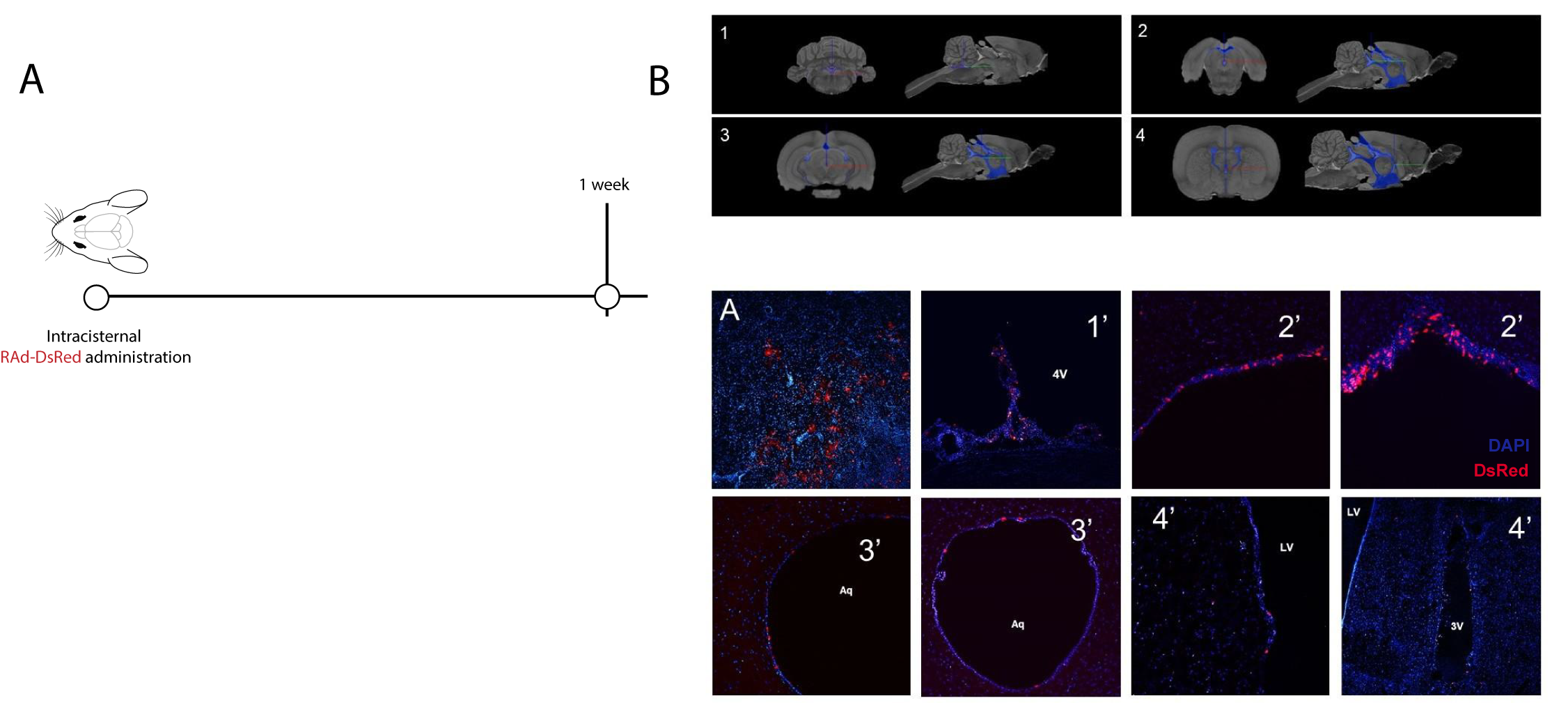

Supplement: SUPPLEMENTARY FIGURE 1 — Assessment of expression of RAd-DsRed vector along the ventricular system after intracisternal administration. (A) Schematic of the experimental design. Two month old rats were intracisternally administered with RAd-DsRed adenoviral vector. After one week, rats were perfused and we assessed the distribution of the expression of DsRed fluorescent protein (Ex λ 583 nm) along the ventricular system. (B) 1, 2, 3 and 4 shows representative images of the rat ventricular system obtained from the Waxholm Space atlas of the Sprague Dawley rat brain (Papp et al., 2014). In panel (B), intra-CPu shows a representative image of the expression of the vector RAd-DsRed locally injected into the striatum. Panel (B) 1’, 2’, 3’ and 4’ show our representative images of the expression of DsRed fluorescent protein along the ventricular system. Aq, Aqueduct; 4V, 4th ventricle; 3V, 3rd ventricle; LV, lateral ventricle. [file Image_1.TIF]

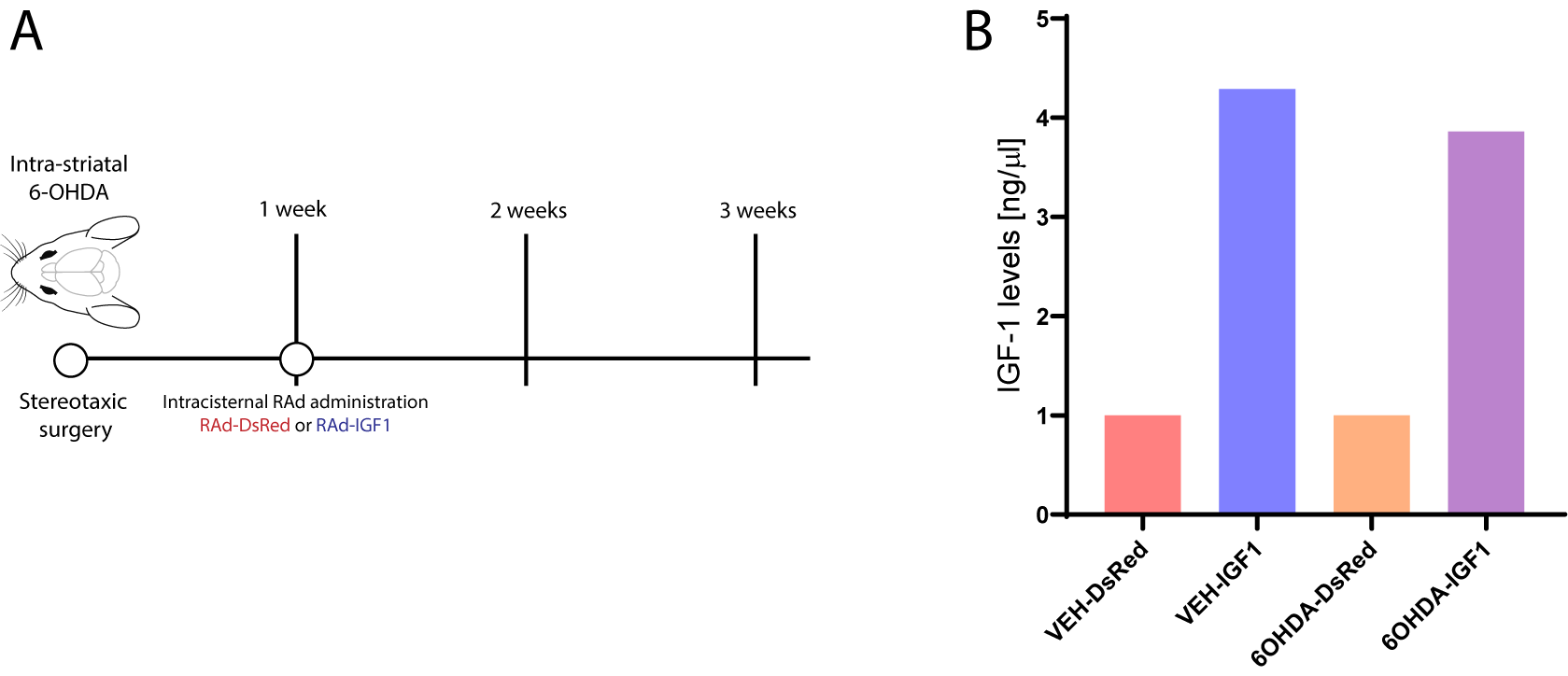

Supplement: SUPPLEMENTARY FIGURE 2 — IGF-1 overexpression assessment. (A) Schematic of the experimental design. IGF-1 concentrations were determined using a commercially available electrochemiluminescence immunoassay (ECLIA) (Elecsys IGF-1, Roche Diagnostics), according to the manufacturer’s instructions. The assay is based on a sandwich immunoassay principle and includes an initial acidification step to dissociate IGF-1 from its binding proteins, followed by the formation of a sandwich complex using a biotinylated monoclonal anti-IGF-1 antibody and a ruthenium-labeled monoclonal anti-IGF-1 antibody. The immune complexes are captured on streptavidin-coated microparticles and quantified by electrochemiluminescence detection. Cerebrospinal fluid samples (CSF) from rats were obtained according to standard laboratory procedures and centrifuged prior to analysis when necessary. Samples were maintained at −20 to −25 °C until IGF-1 quantification. (B) IGF-1 levels in rat CSF. Due to limited sample volume, cerebrospinal fluid samples from animals belonging to the same experimental group were pooled prior to analysis (number of animals per group: VEH-DsRed = 4, VEH-IGF1 = 1, 6OHDA-DsRed = 3, 6OHDA-IGF1 = 4). Concentrations were automatically calculated by the analyzer using a two-point calibration curve and a lot-specific master curve. [file Image_2.TIF]
